# The relative impact of smoking, alcohol use and drug use on general sickness absence among Norwegian employees

**DOI:** 10.1186/s12889-019-6891-1

**Published:** 2019-05-03

**Authors:** Ingeborg Lund, Inger Synnøve Moan, Hilde Marie Erøy Edvardsen

**Affiliations:** 1 Norwegian Institute of Public Health, Department of Alcohol, Tobacco and Drugs, PO Box 222, Skøyen, N-0213 Oslo, Norway; 2 Oslo University Hospital, Department of Forensic Sciences, Section of Drug Abuse Research, Kirkeveien 166, N-0450 Oslo, Norway

**Keywords:** Tobacco, Alcohol, Illegal and medical drugs, Sickness absence

## Abstract

**Background:**

It is well documented that tobacco, alcohol and drug use can be detrimental to health. However, little is known about the relative impact of these factors on sickness absence, and whether the association between use of these substances and sickness absence is different for women and men. The aim of this study was to examine the association between tobacco-, alcohol- and drug use, as well as polydrug use, and sickness absence among Norwegian employees.

**Methods:**

During 2011–2014, 1911 employees in Norway completed a questionnaire about their tobacco, alcohol and drug use habits, their total number of sickness absences during the last 12 months, and the length (no. of days) of their last sick leave. Samples of oral fluid were analysed for illegal and medicinal drugs.

**Results:**

Daily smoking and current use of medical drugs were significantly associated with sickness absence. Employees who were daily smokers also had an increased likelihood of having long and frequent sickness absence. Use of snus (Swedish moist snuff), binge drinking, current use of illegal drugs and polydrug use were not significantly associated with sickness absence. Women and young participants were more likely to report having had sickness absence the past 12 months. However, the associations between daily smoking and medical drug use and sickness absence, respectively, were only statistically significant for men.

**Conclusion:**

According to this study, daily smoking and use of medical drugs are the substance use habits most closely associated with sickness absence. Implications for future research are discussed.

## Background

The Norwegian prevalence of sickness absence amounts to about 6.2% of the total work hours per year [1]. Bearing in mind that differences in measuring practices and workforce-compositions make international comparisons challenging, this is higher than in other European countries [2]. Despite an explicitly stated political goal to reduce it, the Norwegian sickness absence prevalence has remained unchanged for several years [1].

Tobacco-, alcohol- and drug use are lifestyle factors associated with poorer health and reduced mental capacity, and likely to affect sickness absence. While illicit drug use is deemed an important contributor to the global burden of disease [3], cigarette smoking and alcohol use are ranked as some of the most important preventable risk factors [4].

Norwegian tobacco use mainly consists of snus (Swedish moist snuff, a non-combustible oral tobacco product) use and cigarette smoking. In recent years, Norwegian snus use prevalence is approximately similar to smoking prevalence, at about 20% [5, 6]. The detrimental health effects of cigarette smoking are well-established, and involve an increased risk of several forms of cancer [7], increased risk of chronic obstructive pulmonary disease [8] and other respiratory problems [9], and increased risk of cardiovascular disease [9]. Although medical consensus is that snus use is approximately 90% less harmful to health than smoking, studies have provided empirical support for an association between snus use and increased mortality risk in cardiovascular patients, and a modest increase in the risk for heart failure [10]. On the European level, an association has been demonstrated between the use of smokeless tobacco and risk of pancreatic cancer, but among Swedish male snus users, pooled analysis of nine prospective studies found no such association [11].

Even though the majority of tobacco-related harms tend to be chronic and occur after several years of exposure, cardiovascular and respiratory diseases can also result in more acute health problems, e.g. a higher tendency for pneumonia among smokers [12]. The studies that have examined the effect of smoking on sickness absence have found significant associations [13, 14]. However, no study has looked specifically at the association between snus use and sickness absence.

Alcohol use can influence sickness absence through acute effects of episodic heavy drinking, e.g. accidents and hangovers [15], and through the elevated risk of several somatic and psychiatric illnesses associated with chronic heavy drinking [16]. A review study, covering the years 1980 to 2014, concluded that there was empirical support for an association between alcohol use and absence from work, with a stronger effect for short-term than for long-term absence [17].

Most Norwegian studies addressing the association between alcohol use and sickness absence have looked at self-reported absence specifically ascribed to alcohol use [18, 19]. To our knowledge, three studies have examined the link between alcohol use and general sickness absence in Norway. While two studies found a significant relationship between alcohol use and registered sickness absence [20, 21], one study found empirical support for an association between high alcohol intake and self-reported sickness absence [22].

Despite undisputable negative health effects of psychoactive and medical drugs use [4], knowledge about potential effects on sickness absence is limited. In one study, increased absenteeism was found among users of illicit drugs [23], while in another, an increased likelihood of sickness absence was found among workers who reported using cannabis [24]. Furthermore, one study reported a high frequency of sickness absence in some workers with medication-overuse headaches [25].

It is conceivable that polydrug use, i.e. combination use of several substances, could have disproportionately stronger health consequences, and therefore a stronger effect on sickness absence, than the sum of single drug uses would indicate [26]. Several studies have demonstrated that polydrug use has a negative impact on health, and more so simultaneous polydrug use (i.e., the use of more than 1 drug at the same time) than concurrent polydrug use (i.e., various drugs used on separate occasions) (see e.g. McCabe et al. [26]).

While a recent study analyzed the relationship between obesity, low physical activity, smoking and alcohol consumption, and diagnosis-specific sickness absence [27], we were not able to identify any studies examining the relative effect of several substances on general sickness absence. Furthermore, to our knowledge, no previous study have examined whether the association between use of alcohol, tobacco, or drugs and general sickness absence differs between women and men. In many western countries, including Norway, women have a higher sickness absence rate than men [28]. However, absence specifically ascribed to use of alcohol and illegal drugs is more common among men than women [17].

The aim of the current study was to investigate the associations between the use of tobacco, alcohol, and illegal and medical drugs and general sickness absence among Norwegian employees. Additionally, we aimed to study if these associations differed for men and women.

## Methods

Data from 22 Norwegian workplaces within five different business areas was collected during 2011–2014. Altogether 1911 employees (92% participation rate) completed a questionnaire on substance use habits (tobacco, alcohol, medical and illegal drugs), and last year sickness absence. They also provided a sample of oral fluid for analyses of illegal and medical drug content. Participating business areas (no of workplaces/N) included health care (12/917), industry (2/254), restaurants (5/131), media (1/152), and finance (2/457). A predominance of women in the largest branch, health care, was reflected in a prevalence of 60.9% women in the total sample. Age was only asked about in brackets, and ranged from younger than 30 to older than 60. The age distribution differed by business area, with people working in bars and restaurants tending to be younger, and people working in the industry slightly older, than the sample average of approximately 40 years. Table 1 gives a descriptive summary of the sample. A detailed description of other questions included in the questionnaire can be found in Edvardsen et al. [19]. The study was approved by the Regional Committee for Medical and Health Research Ethics.

**Table 1:** Demographic and sickness absence description of sample

	Health	Industry	Restaurants	Media	Finance	Total
N	917	254	131	152	457	1911
Age	%	%	%	%	%	
under 30	15.0	12.2	79.2	25.0	9.5	18.5
30–39	27.2	15.4	16.9	31.6	28.5	25.6
40–49	27.1	31.6	3.1	15.8	25.0	24.6
50–59	22.4	29.6	0.0	21.1	28.3	23.1
60+	8.3	11.3	0.8	6.6	8.6	8.1
Sex						
Male	18.9	80.0	51.9	48.7	50.6	60.9
Female	81.1	20.0	48.1	51.3	49.4	39.1
Education						
Middle school	2.0	9.1	8.4	4.6	0.7	3.2
High School	19.6	53.9	48.1	14.5	14.7	24.5
College	71.3	28.4	40.5	74.3	80.5	65.9
unreported	7.1	8.7	3.1	6.6	4.2	6.3
No of sickness absences last 12 months						
none	26.9	33.9	36.7	27.0	34.0	30.2
1 or 2	51.7	56.2	49.2	57.2	51.9	52.6
3 or more	21.5	10.0	14.1	15.8	14.1	17.2
No of days last sickness absence						
one day or less	58.4	57.6	62.2	56.6	62.6	59.4
2 or 3 days	25.2	26.0	24.4	35.5	26.0	26.3
more than three days	16.4	16.4	13.4	7.9	11.4	14.3

To identify those with a higher occurrence of sickness absences, the variable was recalculated into a dummy variable, with categories representing (0) less than three, and (1) three or more, sickness absences during the last 12 months

### Measures

#### Outcome variables

##### Sickness absence

Self-reported number of sickness absences in the last 12 months showed that 30% of the sample had no last year sickness absence, while 83% reported less than three (including those who had none) (Table 1). The remaining 17% reported from three to more than ten sickness absences.

##### Length of last sickness absence

A dummy variable was created to identify the 14% of respondents who reported a most recent sickness absence lasting longer than three days (1). The three-day limit has a strong position in the Norwegian labour market, as three days or shorter absences do not require a doctor’s attestation of illness. One might therefore construe sickness absences extending three days as an indication of more serious health problems.

##### High-level absence group

The distribution of sickness absence is highly skewed, with a small group of employees accounting for a large proportion of the absence [29]. A high-level absence group was defined as employees reporting both a minimum of 3 sickness absences and a last sickness absence that lasted more than 3 days, i.e. around 5% (*N* = 75) of the sample. To separate this group from the rest, a dummy variable was calculated, with categories representing both requirements fulfilled (1), vs. all others (0). Individuals who reported no sick leaves last 12 months and proceeded to not respond to the question of length, was coded as zeros.

#### Substance use habits

*Smoking* was coded as daily smoking (13.7%), occasional smoking (10.0%), former smoking (28.7%) or never smoking (47.6%).

##### Current daily snus use

In the sample, 7.5% reporting daily, 4.6% occasional and 5.6% former snus use. As research have suggested that occasional and former snus use is not associated with worse self-reported general health [30], a dummy variable was created for current daily snus use (1) vs. all others (0). The all other group thus includes occasional, non- and never-snus users.

##### Weekly binge drinking

Although 94% of the respondents were current users of alcohol, the patterns of use varied greatly. About 36% had drunk alcohol weekly or more often during the last year, while around 4% reported drinking to intoxication at least weekly. The dummy variable *Weekly binge drinking* separates all who reported to have drunk to intoxication at least once a week (1) from those who reported less frequent intoxication episodes (0).

##### Current illegal drug use

The employees in the sample have previously been found to under-report their use of illegal drugs [19]. To reduce the effect of this under-reporting, we chose to include both self-reported data and data from drug testing of oral fluid samples [19]; drug findings in oral fluid reflects in most cases drug intake during past two days. A dummy variable was constructed to indicate last two days self-reported or positive oral fluid-based use of illegal drugs, including cannabis, amphetamine, cocaine, heroin, GHB and LSD (1), vs. no current illegal drug use (0).

##### Current medical drug use

Use of medical drugs was also under-reported by these respondents [19]. A similar combination of self-reported current use and positive test of the oral fluid (use last two days) was used to construct a dummy variable indicating current use of medical drugs (1) vs. no such current use (0). The types of drugs incorporated in the measurement were prescriptive drugs with a potential sedative effect, including anxiety reducing medication, sleep medication, migraine medication, and strong painkillers. No information exists on whether or not the respondents had prescriptions for the drugs.

##### Poly- and dual drug use

Three separate dummy variables were constructed to indicate (1) those who both smoked and drank to intoxication at least weekly, (2) those who both smoked and were current users of medical or illegal drugs, and (3) those who drank to intoxication at least weekly and used medical or illegal drugs.

#### Demographic characteristics and random effects

Other variables included in the analyses were *sex*, *age* (in ten year groups), and *education* (middle school, high school, university/college). *Business area* and *workplace* were included as random effects.

### Statistical analyses

Two multilevel logit analyses were performed on the associations between number of sickness absences (Table 3) and length of last sickness absence (not reported in table), and substance use habits. Business area and workplace were entered as random effects, as they significantly affected these associations (LR-test). For the high-level absence group there was no significant random effects, and a bivariate logistic regression was performed for the associations with substance use (Table 4). Bivariate logistic regressions were used also for separate analyses for men and women (not reported in table).

All regressions were adjusted for sex, age, and education.

## Results

Bivariately, the use of medical drugs and daily smoking were significantly associated with a higher occurrence of sickness absence, with the highest occurrence of more than three last year sickness absences found among current medical drug users (Table 2).

**Table 2:** Bivariate associations between tobacco, alcohol, and drug use and last year sickness absence (*N* = 1392–1888)^a^

	Sickness absence more than twice last 12 months	Last sickness absence more than 3 days duration	High-level absence group
Sample (*N* = 1888)	17.21 (1888)	14.33 (1870)	4.01 (1870)
Weekly binge drinking			
Yes	24.05	11.54	3.85
No	17.01	14.37	4.04
medical drugs			
Yes	27.55**	18.75	7.29
no	16.65	14.09	3.83
Illegal drugs			
Yes	12.5	4.17	0.00
No	17.27	14.46	4.06
Daily smoking			
Yes	22.39*	17.72	6.69*
No	16.46	13.80	3.60

Chi Square, *: *p* < 0.05; **: *p* < 0.01

^a^: variation due to item non-response

No substance-use habit was significantly associated with having been away from work for more than three days on the last sickness absence, but daily smoking was significantly associated with being in the high-level absence group. Weekly binge drinking and the use of illegal drugs was not significantly associated with any of the three sickness absence measurements. However, there was a non-significant tendency for weekly binge drinkers to have been absent due to sickness more than twice in the last year.

In adjusted multilevel analysis (Table 3), daily smokers were significantly more likely to have had three or more last year sickness absences (AOR = 1.65, *p* < 0.05). There was no significant difference between occasional, former, and non-smokers. There was a strong association between sickness absence and current use of medical drugs (AOR = 2.36, *p* < 0.01), but no significant associations were found for snus use, weekly binge drinking, current use of illegal drugs, or poly- or dual drug use.

**Table 3:** Factors associated with having had three or more sickness absences in the past 12 months

	AOR	P	95% CI for AOR
Smoking			
Daily	1.65	0.019	1.09–2.50
Occasional	1.19	0.432	0.77–1.84
Former	1.08	0.617	0.79–1.49
Daily snus use	1.18	0.492	0.73–1.90
Weekly binge drinking	1.76	0.147	0.82–3.78
Illegal drug use	0.77	0.737	0.16–3.65
Medical drug use	2.36	0.007	1.27–4.37
Poly- and drug use			
Smoking and drug use	0.88	0.807	0.33–2.40
Smoking and weekly binge drinking	1.26	0.702	0.39–4.05
Drug use and weekly binge drinking	0.39	0.329	0.06–2.58
Male	0.70	0.024	0.52–0.96
Age (ref = younger than 30 yrs)			
30–39 yrs	1.10	0.625	0.76–1.59
40–49 yrs	0.54	0.004	0.36–0.83
50–59 yrs	0.35	0.000	0.22–0.56
60+ yrs	0.36	0.002	0.19–0.69
Education (ref = middle school)			
High school	1.84	0.225	0.69–4.94
University	1.51	0.409	0.57–4.03
Constant	0.14	0.000	0.05–0.42

Multilevel logit regression (mixed effect logistic regression) with sickness absence (dummy variable) as the dependent variable, controlled for random effects of branch and workplace. AOR = Adjusted Odds Ratio

N = 1811, 22 workplaces (var = 0.12, SE = 0.13) within 5 branches (var = 0.14, SE = 0.08), LR-test for sign. Effect of random stratification: *p* < 0.001

Men were less likely to have had three or more last year sickness absences (AOR = 0.70, *p* < .0.05), while employees aged 40 years or more were less likely (40–49 yrs.; AOR = 0.54, p < 0.01; 50–59 yrs.; AOR = 0.35, *p* < 0.001; 60+ yrs.; AOR = 0.36, p < 0.01) than employees younger than 30 years. No significant association was found for education.

Separate analyses for men and women (not reported in table) showed a significant association with sickness absence for daily smoking (AOR = 2.69, *p* < .01, 95% CI [1.33, 5.42]) and medical drugs use (AOR = 3.76, *p* < .01, 95% CI [1.41, 10.05]) in men, but not in women (AOR = 1.31, *p* = 0.29, 95% CI [0.80, 2.16] and AOR = 1.89, *p* = 0.12, 95% CI [0.85,4.21], respectively).

Substance use habits were not associated with the length of the last sickness absence (not reported in table). However, daily smoking was significantly associated with a higher likelihood of being in the high-level absence group (AOR = 2.61, *p* < 01) (Table 4). There were no significant associations for occasional or former smoking, daily snus use, weekly binge drinking, medical drug use or poly- and dual drug use. The only significant sociodemographic factor was sex, with men being less likely than women to be in the high-level absence group (AOR = 0.45, *p* < 0.01).

**Table 4:** Factors^a^ associated with high-level sickness absence

	AOR	P	95% CI for AOR
Smoking (ref = never)			
Daily	2.61	0.008	1.29–5.30
Occasionally	1.07	0.893	0.42–2.70
Former	1.47	0.199	0.82–2.66
Daily snus use	1.61	0.290	0.67–3.88
Weekly binge drinking	1.01	0.991	0.19–5.28
Medical drug use	2.22	0.103	0.85–5.79
Poly- and dual drug use			
Smoke and drugs	0.50	0.451	0.08–3.01
Smoke and drink	0.62	0.714	0.05–7.95
Drink and drugs	1.94	0.599	0.17–22.73
Male	0.45	0.006	0.26–0.79
Constant	0.02	0.000	0.00–0.08

Binary logistic regression with high-level absence as the dependent variable. *N* = 1801. No significant effect of random stratification. *AOR* Adjusted Odds Ratio

^a^ Adjusted also for age and education, which were not significantly associated with the outcome, and removed from table for increased simplicity. Illegal drug use was not included in the analysis as none of the 75 individuals in the high-level absence group had used illegal drugs

## Discussion

This study represents the first attempt to examine the relative impact of tobacco, alcohol and drug use on general sickness absence among Norwegian employees. In multivariate analysis, daily smoking and current use of medical drugs were significantly associated with sickness absence, while snus use, binge drinking and current use of illegal drugs were not. Separate analyses for men and women showed significant associations only for men. Daily smokers also more often had both three or more sickness absences and a last absence of more than three days.

The results indicated no added effect on sickness absence from poly- and dual drug use beyond the effects of the separate drugs. Since poly- and dual drug use is considered as a more risky lifestyle [26], this might be seen as surprising. However, small polydrug user-groups, and lacking information about use patterns and frequency, make these results subject to large uncertainties.

Medical drug use in groups with pre-existing health problems has probably influenced the association between medical drug use and sickness absence, and might be a contributing factor for the observed difference between sexes. Men and women may use different types of medical drugs, and they may use it for different reasons.

Our results suggest that daily smoking is the tobacco habit most detrimental to health for people in the work force, consistent with findings in the global burden of disease estimates. The higher probability among daily smokers to belong to the high-level absence group is in accordance with the more chronic profile of tobacco harms, as compared to harms from alcohol and medical drugs. A positive association found for men but not for women might reflect the tendency for male smokers to smoke more cigarettes [31].

In recent years, snus use has increased among the young, while the prevalence of smoking has declined sharply the last decades [5]. In light of this, the lack of a significant association between sickness absence and daily snus use may possibly point towards reduced tobacco induced sickness absence in the future.

Seeing as positive associations previously have been reported from Norway [17, 22], the lack of a significant association of alcohol use on sickness absence is surprising, particularly with regard to sickness absence frequency. Concerning the results for longer absence spells, a possible explanation can be that alcohol use is more closely associated with short-term absences [17]. With the exception of the last absence, the current data did not include information on absence length, making separation between shorter and longer absences unattainable. This might potentially have attenuated any alcohol-absence association. An additional factor that may have influenced the results is that the current study pertain to general sickness absence, not absence ascribed explicitly to substance use. When asked specifically about substance related absence, 5.3% of the current respondents reported alcohol-related absence, while 1.5% reported absence due to illegal or medical drugs [32]. A review of Norwegian studies found that 1–2% of employees in general, and 8.1% of young employees, report absence related to alcohol use [18].

### Limitations and suggestions for future research

As this is cross-sectional data, no inferences can be made regarding causality. Generalisability to other areas cannot be ascertained due to small sample size, and a limited selection of work places. For the more uncommon habits, it was difficult to achieve sufficient statistical power, and it is likely for example that the lack of an effect on sickness absence from current use of illegal drugs is due to the scarcity of illegal drug users among the respondents. Small group sizes also implies that the differences found between men and women are subject to uncertainty.

The participants may have under-reported their sickness absence, either intentionally or due to recall bias. Additionally, two possible sources of under-reporting of substance use might have weakened the results. Firstly, the most excessive users of alcohol and drugs, who probably experience most of the negative consequences, are less likely to participate in surveys [33]. Secondly, participants often under-report their own alcohol and drug use [33]. In this study, the under-reporting of illegal and medical drug use was partly accounted for by including data from both questionnaires and samples of oral fluid, but this was not possible for last year alcohol use. It is reasonable therefore to assume that the alcohol- and drug-related figures found in this study represents minimum estimates.

For medical drug use, challenges related to measurements and pre-existing health concerns may have affected our results. In order to distinguish between the relative impact of medical drugs and various health problems on sickness absence, future studies should aim to include a broader set of variables. Future studies should also aim to distinguish between simultaneous and concurrent polydrug use, a distinction that was not possible to make in the current data.

## Conclusion

The results from this study suggest that daily smoking and use of medical drugs are the substance use habits most closely associated with general sickness absence. No association was found for snus use, a habit that has not earlier been studied in relation to sickness absence. The lack of associations between sickness absence and alcohol or illegal drug use are likely due to weaknesses in the data. To improve the knowledge base, future studies should include information about general health status, and more details concerning drug use patterns and frequencies.

## Abbreviation

AOR: Adjusted Odds Ratio
